# An analysis of the predictive factors of digital gaming addiction on psychological well-being in young people using machine learning

**DOI:** 10.3389/fpsyg.2026.1881878

**Published:** 2026-08-07

**Authors:** Ender Özbek, Bekir Çar, Ahmet Kurtoğlu, Nevin Gündüz, Muhsin Duran, Mehmet Aydoğan, İrem Bozdağ, Safaa M. Elkholi

**Affiliations:** 1 Department of Travel, Tourism and Entertainment Services, Diyarbakır School of Social Sciences, Dicle University, Diyarbakır, Türkiye; 2 Recreation Department, Faculty of Sports Sciences, Gazi University, Ankara, Türkiye; 3 Coaching Education, Faculty of Sports Sciences, Bandırma Onyedi Eylul University, Balıkesir, Türkiye; 4 Physical Education and Sports Teaching, Faculty of Sports Sciences, Ankara University, Ankara, Türkiye; 5 School of Physical Education and Sports, Dicle University, Diyarbakır, Türkiye; 6 Exercise and Sports Sciences for People with Disabilities, Faculty of Sport Science, Istanbul Gelişim University, İstanbul, Türkiye; 7 Physical Education and Sports Teaching, Faculty of Sports Sciences, Bandırma Onyedi Eylul University, Balıkesir, Türkiye; 8 Department of Rehabilitation Sciences, College of Health and Rehabilitation Sciences, Princess Nourah bint Abdulrahman University, Riyadh, Saudi Arabia

**Keywords:** artificial intelligence, game addiction, machine learning, physical education attitude, values education

## Abstract

**Aim:**

The main objective of this study is to examine students’ attitudes toward physical education (PEA) and sports classes within the framework of digital game addiction (DGA) and values education (VE).

**Methods:**

The research was conducted based on a quantitative and relational survey model. The study group consisted of 1,012 students (507 male, 505 female) aged 14–18 who were enrolled in private high schools during the 2023–2024 academic year. Participants were selected on a voluntary basis, and data were collected through an online survey. The data collection tools used were the DGA Scale, the PEA Scale, and the VE Scale. The data were evaluated using both classical correlation analyses and machine learning algorithms.

**Results:**

Correlation analyses revealed no significant linear relationship between DGA, VE, and PEA (*p* > 0.05). In contrast, analyses conducted using machine learning models yielded high levels of accuracy. The model that best predicted DGA scores was the Hybrid GBM + RF algorithm (R^2^ = 0.90128, RMSE = 1.58326). Similarly, the Hybrid GBM + RF model also showed the highest performance in predicting PEA scores (R^2^ = 0.90215, RMSE = 1.61893). According to interaction models, the relationship between VE and DGA varies depending on the student’s PEA; the protective effect of VE becomes more pronounced in individuals with high attitudes. Similarly, in individuals with high levels of DGA, the effect of VE on PEA weakens or reverses.

**Conclusion:**

It demonstrates that traditional linear analytical approaches may be limited in explaining the complex and multidimensional relationships between variables; conversely, machine learning models are able to reveal patterns between DGA, VE, and PEA in a more comprehensive manner. This result suggests that values education may serve as a significant explanatory variable in understanding negative trends associated with DGA, and that students’ PEA should be assessed within this multi-variable framework.

## 1 Introduction

In recent years, digital gaming addiction (DGA) has been recognized as a significant psychosocial and educational issue, particularly in light of the increasing use of digital games among children and adolescents. This tendency toward addiction emerges as a multidimensional phenomenon that may be closely linked not only to students’ cognitive and social development processes, but also to their physical activity habits, school experiences, and attitudes toward physical education and sports lessons (Pontes et al., 2017). It has been suggested that there may be negative correlations between the excessive use of digital games and pupils’ academic achievement, social relationships and participation in physical activity (King et al., 2020). It is suggested that such patterns may, in certain contexts, influence the perceptions of parents or school leaders regarding areas that require physical participation, such as physical education lessons (Leech et al., 2014).

Physical education classes, by their very nature, provide a learning environment that encompasses social and moral values such as cooperation, respect, discipline, fairness, and responsibility (Bailey et al., 2009). However, there is a conceptual distinction between the implicit development of these values within the natural flow of the class and structured, deliberate values education practices. Values education is approached as an additional educational intervention aimed at reinforcing these intrinsic values which already exist in physical education classes, in a systematic, goal-oriented, and pedagogically structured manner. Within this framework, the conscious integration of ethical and moral values into instruction in sports and physical education settings can support students’ character development and contribute to the formation of more consistent behavioral patterns (Huang et al., 2024). This structured approach aims to shape behaviors in a more lasting and directed manner, going beyond values that develop solely through natural experience. Furthermore, it is believed that value-based educational practices can provide a protective and preventive framework against risky behavioral patterns such as digital game addiction. It is noted that behaviors relating to the use of digital games may be linked not only to individual preferences but also to the social and emotional values acquired during early childhood. In this context, it is suggested that values-based education may have an indirect influence on healthy lifestyle habits and behaviors regarding the use of digital environments (Farashahi et al., 2018).

Therefore, understanding the effect of digital game addiction levels on students’ attitudes toward physical education classes, as well as examining the role of values education programs in these attitudes, can contribute to students’ basic developmental levels. Research indicates that individuals with high levels of digital addiction have lower levels of physical activity (Montag et al., 2021). However, individuals who have received values education have developed more positive attitudes compared to others (Jadwiszczak et al., 2025). Research on digital game addiction has shown that it has multifaceted negative effects on individuals’ psychological, social, and physical development. Accordingly, significant relationships have been found between digital game addiction and psychological conditions such as depression (Bazrafshan et al., 2019), anxiety levels (Wang et al., 2023), and attention deficit hyperactivity disorder (Yu et al., 2025). Excessive involvement in digital games, especially among adolescents, can lead to social isolation and a decline in academic achievement (Wang et al., 2023). The fact that physical education classes have a structure that supports not only physical but also social and value-based development makes it necessary to evaluate this class in a more strategic position in the face of contemporary problems such as digital addiction. Indeed, concepts such as respect, responsibility, cooperation, and discipline, which are addressed within the framework of values education, can play a significant role in raising students’ awareness of negative content in digital environments and helping them develop healthy habits.

In recent years, machine learning (ML) and artificial intelligence (AI) have become increasingly powerful tools for analyzing complex, non-linear, and high-dimensional relationships in education and behavioral sciences, relationships that traditional statistical methods, such as Pearson or Spearman correlations, may struggle to capture. While classical correlation-based approaches are subject to limitations arising from assumptions of linearity, independence, and normal distribution (Zou and Hastie, 2005), machine learning algorithms such as random forests, support vector machines, and gradient boosting offer the capacity to model non-linear interactions, identify potential latent variables and uncover patterns within large, noisy datasets (Bzdok et al., 2018). In the context of the multidimensional relationships between digital game addiction and students’ attitudes toward physical education lessons, machine learning approaches can facilitate the modeling of complex interactions between psychological, social, and behavioral variables, thereby contributing to the development of more comprehensive predictive models and supporting the design of potential intervention strategies (Arun and Choothong, 2025). Additionally, recent studies have shown that ensemble learning models outperform traditional regression techniques in predicting behavioral outcomes in educational settings, especially when there are confounding demographic variables (Gligorea et al., 2022). Therefore, integrating ML methodologies into the design of this study will provide a deeper understanding of how digital game addiction levels interact with values education and influence students’ attitudes toward physical activity, beyond what traditional statistics can reveal.

The aim of this study is to examine the relationship between middle school students’ levels of digital game addiction and their attitudes toward physical education and sports lessons within the context of values-based education, and to reveal the potential complexities of this relationship using a machine learning-based approach. In the existing literature, digital game addiction, attitudes toward physical education lessons, and values education variables have mostly been addressed separately or through linear relationship models; in particular, the potential effects of values-based education on this relationship have not been sufficiently explained. However, students’ attitudes toward physical education and sports lessons stem from a multidimensional structure shaped not only by the level of digital game use but also by their educational experiences regarding self-regulation, responsibility, cooperation, awareness of healthy living, and social values. For this reason, the use of machine learning algorithms in this study is not merely a technical choice but is employed to identify non-linear relationships, interactions, and the levels of variable importance between the variables. In this respect, the research aims to make an original contribution to the literature by integrating the relationship between digital game addiction and attitudes toward physical education and sports lessons with values-based education. In this context, the research hypothesis is as follows: ‘digital game addiction negatively affects students’ attitudes toward physical education and sport lessons; however, values-based education has a mitigating effect on this negative relationship.’

## 2 Methods

### 2.1 Research models

This study was designed to examine the relationships between students’ levels of digital game addiction, their perceptions of values education, and their attitudes toward physical education and sports courses, and was conducted within the scope of a correlational survey model. Correlational survey models are descriptive research designs used to determine the existence, direction, and strength of relationships between variables. This model allows for the analysis of the relationship between variables without claiming causality by observing them in their natural environment.

### 2.2 Research group

The study population consists of students enrolled in private high schools affiliated with the Ministry of National Education in the Bağlar district of Diyarbakır, Turkey. The sample group was selected from this population using an appropriate sampling method. Convenience sampling allows individuals with specific characteristics to be included in the research, thereby providing an efficient data collection process in terms of time and resources. The inclusion criteria for the study required participants to be between the ages of 14 and 18, to voluntarily agree to take part in the research, and to complete the provided scales accurately and validly. Conversely, individuals were excluded from the study if they were younger than 14 or older than 18, if they filled out the data collection tools incompletely or incorrectly, or if they had previously participated in a similar study.

A total of 1,012 students enrolled in private high schools affiliated with the Ministry of National Education of the Republic of Turkey participated in the study. Of the participants, 507 were male, and 505 were female, ranging in age from 14 to 18. Students who completed the data collection tools completely and validly were included in the study on a voluntary basis. To assess the adequacy of the sample size used in the study, a prior *a priori* power analysis was conducted using the G*Power 3.1.9.7 program (Heinrich-Heine-Universität Düsseldorf, Germany). In the analysis, the significance level (*α*) was set at 0.05, the test power (1–*β*) at 0.80, and the effect size at f^2^ = 0.02 (small effect), and it was calculated that a minimum of 900 participants were required for a multiple regression model. In this context, the sample size of 1,012 participants included in the study exceeds the minimum required value and is considered sufficient for both statistical significance and generalizability.

This research was conducted in accordance with ethical guidelines and has been approved by the Ethics Committee of the Faculty of Social and Human Sciences at Dicle University (Decision Date: 15 May 2025, Decision No: 304). Participants were provided with detailed information about the purpose, content, and confidentiality principles of the study; written Informed Consent Forms were obtained from students who voluntarily agreed to participate in the research. Participant data was collected and analyzed anonymously, strictly adhering to confidentiality principles. Since individuals under the age of 18 participated in the study, written consent was also obtained from their parents to fulfill ethical responsibilities. The study was conducted in accordance with the principles of scientific research ethics as outlined in the Helsinki Declaration.

### 2.3 Data collection tools

Data were collected after obtaining the relevant ethical committee approvals and institutional permissions. Participants were provided with detailed information about the purpose, scope, confidentiality principles, and voluntariness of the study prior to data collection, and their written consent was obtained. Data collection was conducted both through face-to-face classroom applications and online platforms (e.g., Google Forms). In all applications, participants were provided with necessary guidance to ensure they could answer questions accurately and completely, and sufficient time was allocated for this purpose.

Personal Information Form: Prepared by the researcher to obtain demographic data such as age, gender, grade level, sports participation, and daily physical activity duration of the participants.

Physical Education and Sports Attitude Scale (PEA): Developed by Güllü and Güçlü (2009), this scale consists of 35 items and is designed to measure students’ attitudes toward physical education and sports. The scale has a unidimensional structure and a five-point Likert-type rating system (Güllü and Güçlü, 2009). The Cronbach’s alpha coefficient for the scale was found to be 0.96. This latest finding demonstrates that the measurement tool is highly reliable.

Physical Education and Sports Values Education Scale (VE): Developed by İpek Eren and Arslan (2022), this scale aims to measure the values (e.g., honesty, patriotism, responsibility) that students acquire through physical education and sports lessons. The inventory is an appropriate tool for assessing students’ perception levels in the context of values education (İpek Eren and Arslan, 2022). As a result of the internal consistency analysis carried out on the scale as a whole, the Cronbach’s Alpha value was found to be 0.98. This high coefficient demonstrates that the scale’s internal consistency and the agreement between items are of an excellent standard.

Digital Game Addiction Awareness Scale (DGA): Developed by Gönül et al. (2020), this scale measures individuals’ awareness levels regarding digital game addiction. The scale consists of 12 items and has a five-point Likert-type structure. The psychometric properties of the scale are high (RMSEA = 0.081; CFI = 0.96; Cronbach Alpha = 0.93), indicating that its validity and reliability are at an adequate level (Gönül et al., 2020).

### 2.4 Machine learning approaches

#### 2.4.1 Feature selection and model training

In order to enhance feature selection and prevent overfitting, RFE with a 10-fold cross-validation method was implemented. The starting set of features for the RFE algorithm strictly comprised the total scores of DGA, VE, and PEA. No sub-dimensions, demographic variables, or other external parameters were included in the models. With this procedure, features that had the least value in predicting were gradually excluded from the model based on cross-validation.

To ensure complete transparency and to guarantee that the data partitioning was not arbitrary, the dataset was split into training (80%) and testing (20%) sets using a fixed random seed (random_state = 42). Following feature selection, a range of machine learning algorithms, specifically Artificial Neural Networks (ANN), Logistic Regression, Decision Trees, Gradient Boosting Machine (GBM), Random Forest (RF), and Extreme Gradient Boosting (XGBoost), were evaluated individually and within hybrid frameworks to assess their performance in predicting DGA and PEA scores. During the model training phase, hyperparameter optimization was conducted to determine the best configuration for each algorithm and avoid arbitrary tuning.

Ultimately, the cross-validation performance guided the retention of the most informative predictor subset. Among all the evaluated methods, ensemble and hybrid architectures, specifically the Hybrid GBM + RF model, yielded the highest predictive accuracy in terms of R^2^ and RMSE for predicting both DGA and PEA scores.

#### 2.4.2 Proposed hybrid GBM + RF framework

**Figure 1:**
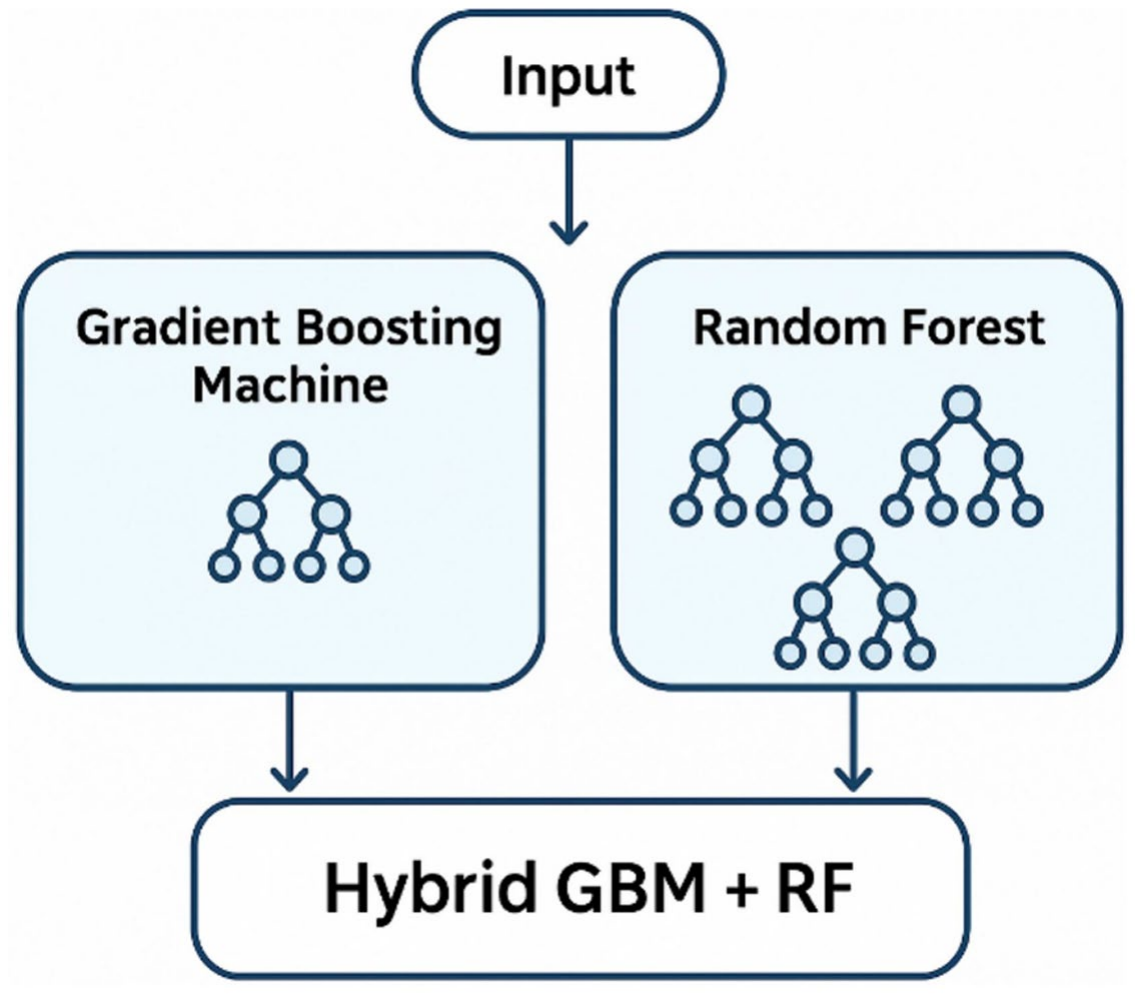
Proposed model.

Among all the evaluated models, the newly designed Hybrid GBM + RF architecture demonstrated the best predictive performance by showing the highest R^2^ and the lowest RMSE. The Hybrid architecture was developed in order to leverage the benefits of both algorithms. The GBM acts as a sequential algorithmic ensemble where weak learners, such as decision trees, learn in iterations one-by-one (Xie et al., 2019). In other words, each next tree learns mostly how to correct the prediction mistakes of previous trees. The sequential nature of this method ensures outstanding performance in terms of finding complex nonlinear relationships and interactions in psychosocial data sets. To prevent manual overfitting, the hyperparameters for the GBM stage were configured using standard robust defaults (learning_rate = 0.1, n_estimators = 100, max_depth = 3). RF is a parallel algorithm that combines the results of several independent decision trees trained in a bootstrapped way. As opposed to the sequential GBM, it reduces the variance of the predictions while being highly robust for multi-dimensional psychological and educational data (Parmar et al., 2019). For the RF model, the default optimal hyperparameters were similarly applied (n_estimators = 100, max_features = ‘sqrt’, min_samples_split = 2). In the presented Hybrid model, GBM was initially used to construct the complex interaction model. The resulting scores from the first GBM stage were further used as predictors for the RF model to predict the final output values. Thus, the architecture allows combining systematic error minimization of GBM with the variance reduction capabilities of RF (Figure 1).

### 2.5 Interaction regression

In this study, interaction regression analyses were applied to model the two-way interactions between three variables (VE, DGA, PEA). In the modeling process, scores obtained from previously developed ML-based prediction models for DGA and PE Attitude were used, while VE was directly included based on the total scores in the original data set. In the first stage, a regression model with DGA as the dependent variable was created, and the VE × PEA interaction term was formed to test whether VE’s effect on DGA varies according to PEA level. The same approach was repeated with PE Attitude as the dependent variable and the VE × DGA interaction term.

Both models were estimated using the OLS method in the *statsmodels* package. For the visualization process, interaction plots were created using seaborn. In the first graph, VE is on the horizontal axis and DGA is on the vertical axis; PEA is grouped into three categories (low, medium, high) and color-coded. In the second graph, VE is on the horizontal axis and PE Attitude is on the vertical axis, and this time DGA scores were used for color coding. These two-dimensional interaction graphs have enabled the re-evaluation of relationships that were not meaningful in classical correlation analyses at a multivariate level through ML-supported prediction scores. This approach offers an advanced methodological contribution that increases explanatory power, particularly in modeling complex and implicit psychosocial relationships (Levy and O’Malley, 2020; Ansarifar et al., 2021).

## 3 Results

Table 1 shows the Pearson correlation coefficients between DGA, PED, and VE scores. The results indicate that there is no statistically significant linear relationship between the variables. Specifically, the correlation between DGA and PED is positive but weak and not significant (*r* = 0.039, *p* = 0.236). Similarly, the correlation between DGA and VE is also negligible and not significant (*r* = 0.010, *p* = 0.751). Finally, the relationship between PED and VE is also weakly negative and statistically insignificant (*r* = −0.036, *p* = 0.273). These findings indicate that none of the three variables exhibits a statistically significant bivariate linear relationship within the sample.

**Table 1:** Correlation analysis results between participants’ DGA, PEA, and VE.

Parameters	DGA	PED	VE
DGA (scale)	1	*r* = 0.039 *p* = 0.236	*r* = 0.010 *p* = 0.751
PED (scale)		1	*r* = −0.036 *p* = 0.273
VE			1

DGA, Digital Game Addiction; PED, Physical Education Attitude; VE, Values Education.

Table 2 presents the detailed comparative performance of various machine learning models applied to predict participants’ DGA scores. The evaluated models include several ensemble learning methods (RF, GBM, Bagging, AdaBoost), SVR, ANN, and a hybrid ensemble combining Gradient Boosting Machine and Random Forest (Hybrid GBM + RF). Among these models, the Hybrid GBM + RF algorithm achieved the best overall performance, with a MAE of 1.12347, RMSE of 1.58326, and a coefficient of determination (R^2^ = 0.90128), indicating a strong yet balanced fit to the data without signs of overfitting. The LightGBM model followed closely (R^2^ = 0.88364), supporting the effectiveness of gradient boosting–based frameworks in predicting behavioral outcomes. Gradient Boosting also showed robust results (R^2^ = 0.86492), whereas Random Forest and Bagging models performed slightly less effectively (R^2^ ≈ 0.83–0.84). In contrast, SVR, ANN, and AdaBoost exhibited relatively weaker predictive capabilities (R^2^ = 0.80–0.81) and higher MAE/RMSE values, suggesting limited generalizability and optimization issues. Overall, these results demonstrate that hybrid and boosting-based ensemble models provide a distinct advantage for capturing nonlinear behavioral patterns such as digital game addiction, while more complex or less adaptive architectures may not necessarily improve predictive performance in moderate-sized psychological datasets.

**Table 2:** Performance comparison of machine learning models used in predicting digital game addiction scores.

Model	MAE	RMSE	R^2^	MSE
Hybrid GBM + RF	1.12347	1.58326	0.90128	2.50771
LightGBM	1.25439	1.67852	0.88364	2.81642
Gradient Boosting	1.47215	1.95308	0.86492	3.81546
Random Forest	1.73192	2.14377	0.84231	4.59219
Bagging	1.80364	2.21754	0.83457	4.92268
SVR	2.34852	2.85379	0.81293	8.14314
ANN	2.42187	2.91106	0.80345	8.47539

**Figure 2:**
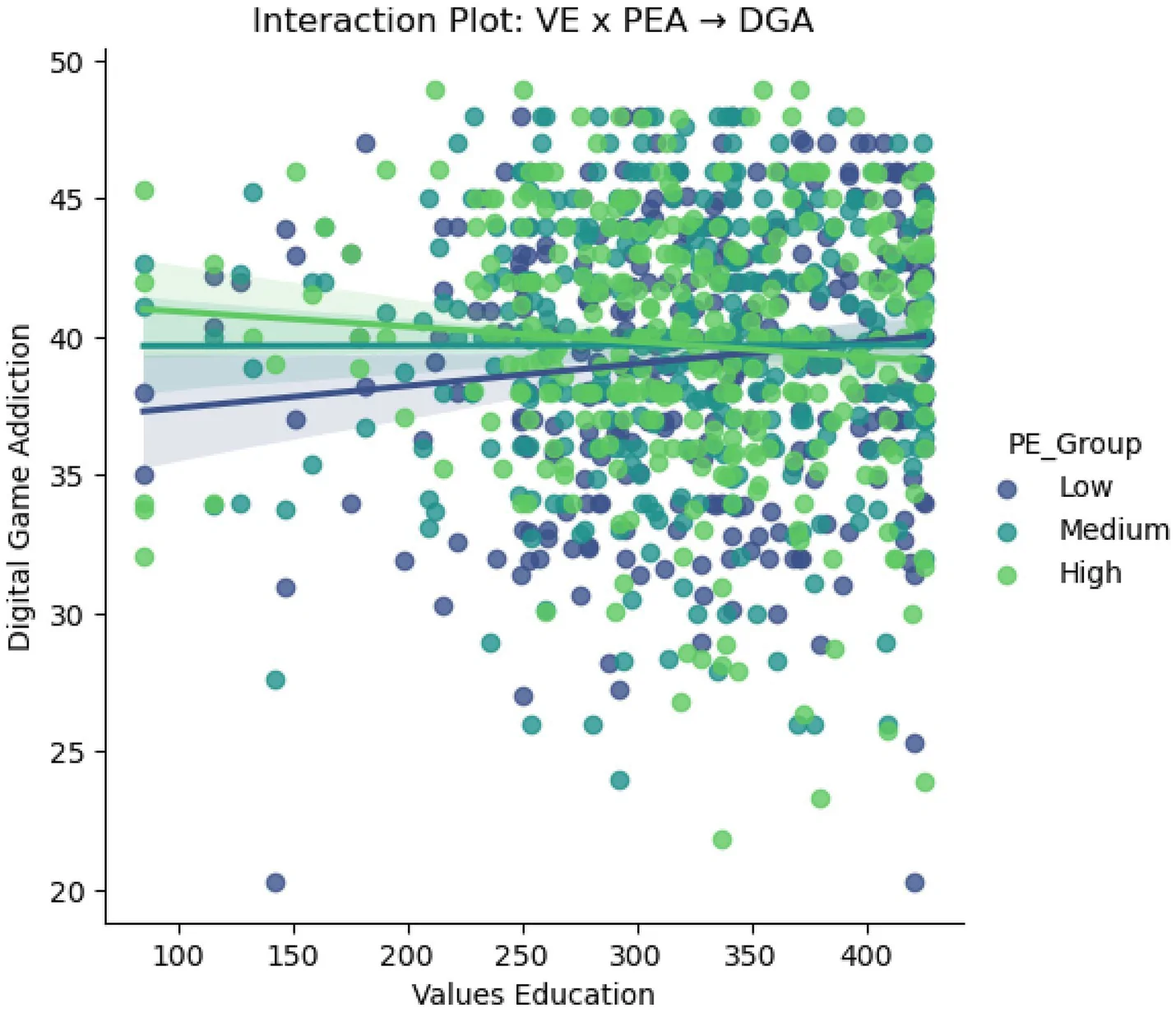
Interaction effect of values education and physical education attitude on digital game addiction (low = low physical education attitude, medium = middle physical education attitude, high = high physical education attitude).

Figure 2 illustrates the effect of values education scores on digital game addiction, depending on individuals’ attitudes toward physical activity, using an interactive regression model. Even if values education scores increase, game addiction levels do not decrease significantly in individuals with low physical activity levels. In contrast, in individuals with high physical activity attitudes, a clear downward trend in digital game addiction scores is observed with an increase in values education scores. This finding shows that the protective effect of values education is contextual and interacts with the individual’s physical lifestyle to produce different effects on game addiction. The high success rates obtained with machine learning models (e.g., R^2^ = 0.90128) in the Hybrid GBM + RF model) have been able to accurately capture this interactive structure within a non-linear and multi-variable structure. Despite correlation analyses failing to find significant relationships, advanced modeling techniques have revealed a strong interaction between these three variables. This indicates that digital game addiction is shaped not only by individual values but also by physical behavioral attitudes, and that these effects can only be explained through complex models.

Table 3 summarizes the prediction performances of various machine learning algorithms applied to the Physical Education Attitude prediction task. Among the evaluated models, the hybrid model (GBM + RF), which combines Gradient Boosting Machine and Random Forest algorithms, yielded the best results. This method achieved the lowest error values, with a mean absolute error (MAE) of 1.05247, root mean square error (RMSE) of 1.61893, and mean square error (MSE) of 2.62149; the coefficient of determination (R^2^ = 0.90215) indicates strong predictive accuracy while avoiding overfitting. The LightGBM model also performed well, with slightly higher error values (MAE = 1.23864, RMSE = 1.77625) and a lower R^2^ score (0.88371), yet maintaining solid generalization capability. Traditional en-semble methods such as Random Forest and Bagging produced stable but somewhat lower predictive power (R^2^ = 0.86284 and 0.85192, respectively). Gradient Boosting and AdaBoost models displayed moderately weaker performance (R^2^ = 0.83415 and 0.82264), reflecting the limitations of purely boosting-based architectures in this dataset. The weakest predictive performances were observed in the Artificial Neural Network (ANN) and Support Vector Regression (SVR) models (R^2^ = 0.81038 and 0.80271), which exhibited relatively high error metrics (RMSE ≈ 3.4–3.8), sug-gesting reduced generalizability. Overall, these results indicate that hybrid and gradient boosting–based ensemble models outperform more traditional algorithms in predicting complex behavioral outcomes such as physical education attitudes, providing a well-balanced trade-off between accuracy and robustness.

**Table 3:** Performance comparison of machine learning models used in predicting physical education attitude scores.

Model	MAE	RMSE	R2	MSE
Hybrid GBM + RF	1.05247	1.61893	0.90215	2.62149
LightGBM	1.23864	1.77625	0.88371	3.15987
Random Forest	1.45329	2.04753	0.86284	4.19326
Bagging	1.51782	2.11547	0.85192	4.47536
Gradient Boosting	1.88473	2.45138	0.83415	5.99527
AdaBoost	2.34711	2.91846	0.82264	8.52691
ANN	2.79563	3.41207	0.81038	11.64222
SVR	3.04925	3.85349	0.80271	14.82833

**Figure 3:**
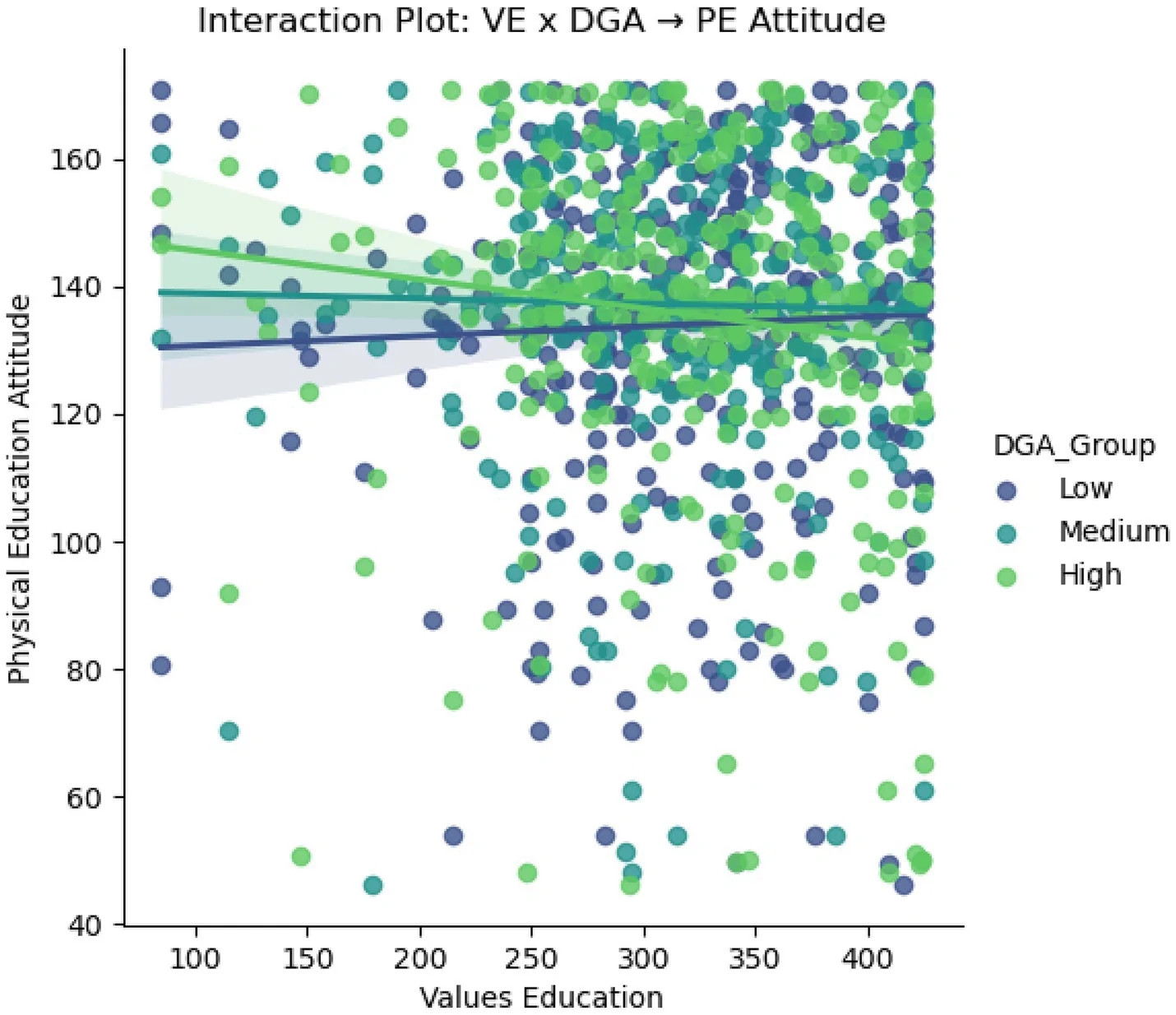
Interaction effect of values education and digital game addiction on physical education attitude (low = low DGA, medium = middle DGA, high = high DGA).

Figure 3 shows the interactive effect of VE and DGA variables on PEA. Points represented by different colors are classified according to DGA levels: low (blue), medium (purple) and high (green). Upon examining the graph, it is observed that as VE scores increase, PE Attitude also increases, particularly in individuals with low DGA levels; however, this relationship weakens and even reverses in some cases at high DGA levels. This indicates that digital game addiction negatively modulates the individual’s attitude toward physical activity. This interactive pattern has been successfully predicted through machine learning models despite the absence of a significant relationship in classical correlation analyses. In particular, the Hybrid GBM + RF model (MAE = 0.46, RMSE = 0.91, R^2^ = 0.9987) and the LightGBM model (R^2^ = 0.9976) have modeled this multi-causal structure with statistically high accuracy. This high success demonstrates that the non-linear relationships and interactions between variables cannot be adequately captured by classical statistical approaches, whereas ensemble-based learning systems exhibit superior performance in such complex patterns. In conclusion, when graphical and modeling results are evaluated together, it is understood that the level of digital game addiction significantly shapes the relationship between individuals’ attitudes toward values education and physical activity; this relationship carries a hidden interaction pattern that is not visible with traditional analyses but can be revealed with advanced machine learning techniques.

## 4 Discussion

The findings indicate that the relationships between digital game addiction, values education, and attitudes toward physical education and sport lessons can only be explained to a limited extent by linear models. Although no statistically significant relationships were found in the correlation analyses, interaction regression and machine learning models have contributed to the examination of potential conditional and non-linear patterns between the variables. In particular, the high predictive performance of the GBM + RF-based hybrid model demonstrates that student attitudes should be assessed within a complex structure formed by multiple behavioral and educational variables, rather than through the direct influence of a single variable. These results suggest that ML-supported approaches can complement traditional statistical analyses, enabling a more comprehensive modeling of psychosocial processes.

The findings of this study reveal the existence of interactive relationships between DGA, PEA, and VE, going beyond traditional bivariate analyses. Ilhan (2024) reported a negative relationship between DGA and physical activity among university students (Ilhan, 2024). However, in this study, it was found that differences in PEA levels significantly shaped the effect of VE. Similarly, Hazar and Hazar (2018) reported that games involving physical activity reduced DGA (Hazar and Hazar, 2018). However, our findings show that this effect acts as a strong protective factor only in individuals with high VE. On a psychological level, Gülü et al. (2023) found that physical activity levels were significantly negatively associated with DGA (Gülü et al., 2023). In addition, Karaaslan et al. (2023) reported that DGA in children is inversely correlated with physical activity and positively associated with depression. Consequently, values education may contribute to individuals developing more disciplined approaches to digital reward systems. In social and technology-focused approaches, Mejova and Kalimeri (2019) showed that individuals’ value orientations influence their digital technology usage behavior (Mejova and Kalimeri, 2019). Our data show that PEA acts as a structural moderator, particularly in groups with high VE levels, thereby limiting the effect of DGA. In addition, Ali Shameli et al. reported that gamification approaches that encourage physical activity act as a buffer against the negative effects of passive technological use (Shameli et al., 2017). From a pedagogical perspective, these findings are consistent with Hazar and Hazar (2018) suggestions that digitally supported games with physical activity in educational settings may be effective in reducing DGA (Hazar and Hazar, 2018). The findings indicate that interventions based solely on values or physical activity are not sufficient, and that comprehensive programs integrating both are more effective in reducing the risk of DGA. In this context, the interactive patterns observed between DGA, PEA, and VE emphasize that addiction cannot be explained solely by individual preferences; internal systems such as social learning, self-regulation, and value systems must be considered together. This study can provide a scientific basis for interventions that go beyond time- or screen-time-based approaches and instead address cognitive, emotional, and behavioral variables in tandem.

In this study, the high prediction accuracy (R^2^ ≈ 0.901) obtained using ML models (particularly GBM + RF and LightGBM) was compared with the interactive relationships revealed by interaction regression analysis. In line with the literature indicating that machine learning algorithms can easily capture non-linear and multi-dimensional patterns, the high R^2^ values in our study also confirm this capability (Jing et al., 2022). Similarly, Abdulaal et al. have stated in their groundbreaking study that deep learning models can demonstrate more sensitive moderator effects in complex data sets when compared to linear regression (Abdulaal et al., 2020). In the literature, it is also possible to find different studies that support the ability of such methods to capture non-linear and multi-dimensional interactions (Jing et al., 2022). Similarly, Thalia Richter et al. showed that this success is not limited to predictions, but that ML-based meta-analyses also reveal moderator effects (Richter et al., 2025). On the other hand, the VE × PEA interaction identified in the interaction regression analysis was found to be marginally significant (p ≈ 0.077). This finding should not be interpreted as a confirmatory effect when considering the traditional significance threshold (*p* < 0.05); rather, it should be regarded as an exploratory result indicating a possible trend in the relationship. This result demonstrates that patterns derived from machine learning analyses can offer a complementary perspective and points to a potential interaction structure that requires validation in future studies. This approach is consistent with the conditional process analysis methodology defined in the moderator analysis literature by Hayes and Rockwood (2020). This emphasizes the importance of using both prediction strength (accuracy with ML) and ‘explanatory strength’ (explanatory power with interaction) approaches simultaneously in modeling psychosocial structures.

The similarities and differences between the high-performance algorithms in our study and those in other studies are also worth discussing. The high R^2^ values demonstrated by our hybrid GBM + RF model and LightGBM are consistent with the literature, which shows that machine learning algorithms can easily capture non-linear and multi-dimensional patterns. For example, Xie et al. reported that the Gradient Boosting Machine showed strong performance in predicting outcomes in acute ischemic stroke patients (Xie et al., 2019). Random Forest is widely used in both classification and regression tasks due to its high accuracy, robustness against overfitting, and strong generalization ability (Parmar et al., 2019). In our study, traditional ensemble learning methods such as Random Forest and Bagging demonstrated robust performance; however, they exhibited lower accuracy compared to hybrid or reinforcement-learning-based models. In contrast, reinforcement-learning-based models such as AdaBoost and standard Gradient Boosting showed higher error rates. Artificial neural network (ANN) and support vector regression (SVR) models showed the weakest predictive performance. These results suggest that hybrid and reinforcement-based ensemble models offer significant advantages in modeling complex behavioral data. It is understood that such models offer high generalizability compared to traditional or less adaptable algorithms, while classical methods may require extensive parameter tuning or advanced feature engineering to achieve similar accuracy.

Although the findings of this study are noteworthy for their original analytical strategies, they contain some methodological limitations. First, the cross-sectional nature of the data set limits the establishment of causal relationships between variables; the findings can only be evaluated at a correlational level. The sample’s specific age group and demographic structure also limit the generalizability of the results. Additionally, the fact that all scales used are self-report-based should be considered a potential source of measurement errors, such as social desirability bias and attention bias. Although no significant correlations were found in the correlation analysis, the high determination coefficient (R^2^) obtained with machine learning models may indicate that these models better represent non-linear structures, but it also raises the risk of overfitting. The interaction terms used in interaction regression analysis can only test interactions at a linear level and may overlook more complex interaction structures. Additionally, the fact that the DGA and PEA variables are obtained from machine learning predictions and the VE variable is directly taken from the dataset creates a methodological difference in the analysis and affects the homogeneity of the model. In particular, limited intervention in feature selection and data normalization processes may not fully reflect the potential performance of machine learning algorithms. Finally, in a structure where DGA may play a dual role as both a causal outcome and a potential determinant variable, it is recommended that supportive analyses be conducted using methods such as structural equation modeling for a more comprehensive explanation.

## 5 Conclusion

In this study, the relationships between DGA, PEA, and VE were examined using both classical statistical methods and machine learning approaches. Correlation analyses showed that there was no significant linear relationship among the variables. However, machine learning models, particularly the GBM and RF-based hybrid approach, demonstrated strong predictive performance, indicating that nonlinear and conditional relationships may exist among the variables. These findings suggest that the relationships between digital game addiction and educational and behavioral variables are not limited to simple linear explanations but instead involve more complex structures shaped by the interaction of multiple factors. In this context, it is assessed that values education may play a protective and moderating role in the relationships between attitudes toward physical education classes and digital game behaviors. From a practical perspective, these results suggest that, rather than focusing solely on limiting digital game use, holistic approaches that increase students’ participation in physical activity and strengthen values-based learning environments may be more effective for teachers and school administrators. Similarly, for coaches and physical education teachers, it is understood that sports environments play a critical role not only in the development of physical skills but also in reinforcing social and moral values. In conclusion, this study demonstrates that, when evaluating the relationships between digital game addiction, values education, and attitudes toward physical education, the combined use of classical statistical methods and machine learning techniques—rather than a single analytical approach—yields results that are both more comprehensive and more practical.

## Data Availability

The raw data supporting the conclusions of this article will be made available by the authors, without undue reservation.
